# Spatial Autocorrelation Can Generate Stronger Correlations between Range Size and Climatic Niches Than the Biological Signal — A Demonstration Using Bird and Mammal Range Maps

**DOI:** 10.1371/journal.pone.0166243

**Published:** 2016-11-17

**Authors:** Véronique Boucher-Lalonde, David J. Currie

**Affiliations:** Department of Biology, University of Ottawa, Ottawa, Ontario, Canada; Helmholtz Zentrum Munchen Deutsches Forschungszentrum fur Umwelt und Gesundheit, GERMANY

## Abstract

Species’ geographic ranges could primarily be physiological tolerances drawn in space. Alternatively, geographic ranges could be only broadly constrained by physiological climatic tolerances: there could generally be much more proximate constraints on species’ ranges (dispersal limitation, biotic interactions, etc.) such that species often occupy a small and unpredictable subset of tolerable climates. In the literature, species’ climatic tolerances are typically estimated from the set of conditions observed within their geographic range. Using this method, studies have concluded that broader climatic niches permit larger ranges. Similarly, other studies have investigated the biological causes of incomplete range filling. But, when climatic constraints are measured directly from species’ ranges, are correlations between species’ range size and climate necessarily consistent with a causal link? We evaluated the extent to which variation in range size among 3277 bird and 1659 mammal species occurring in the Americas is statistically related to characteristics of species’ realized climatic niches. We then compared how these relationships differed from the ones expected in the absence of a causal link. We used a null model that randomizes the predictor variables (climate), while retaining their broad spatial autocorrelation structure, thereby removing any causal relationship between range size and climate. We found that, although range size is strongly positively related to climatic niche breadth, range filling and, to a lesser extent, niche position in nature, the observed relationships are not always stronger than expected from spatial autocorrelation alone. Thus, we conclude that equally strong relationships between range size and climate would result from any processes causing ranges to be highly spatially autocorrelated.

## Introduction

Species’ geographic ranges have long been thought to be strongly limited by their tolerances of climate [1]. Temperature and water availability, in particular, are generally thought to be important axes of the fundamental niche [2], which encompasses all environmental conditions in which a species can survive [3]. The realized niche, i.e. the environmental conditions experienced within the species’ range, is set by the fundamental niche and reduced, or expanded, by dispersal, biotic interactions and the available set of conditions [4]. At macro-scales and coarse grains, climate is generally assumed to be an important range determinant, with range limits close to the species’ climatic tolerances [5–7].

Strong climatic constraints on individual species’ ranges are also thought to lead to higher-order patterns in nature such as the richness–climate relationship [8, 9] and inter-specific variations in range size [10, 11]. In particular, species that are able to tolerate broader environmental conditions are hypothesized to be more widely distributed: niche breadth positively affects range size [11, 12] through an increase in species’ potential range size. Biotic interactions (e.g., greater parasitism in warm climates) and dispersal limitation (e.g., colonization lags following glaciations) are hypothesized to exclude species from occurring in all suitable climatic conditions, such that species whose biology allows them to fill a greater proportion of their fundamental niche should have larger ranges [13–16]. Last, niche position, i.e. where species’ niche occurs along a given climatic gradient, has also been hypothesized to affect range size through various mechanisms [17–19].

Tests of these hypotheses relating range size to species’ biological characteristics typically rely on measuring climatic tolerances from observed geographic ranges. Early and Sax [20] noted that “the use of species’ distributions to measure species’ environmental tolerances is one of the most fundamental techniques in biogeography”. Physiological climatic tolerances are costly and time-consuming to measure (but see [9, 21, 22]) and in-lab measurements are not necessarily transferable to natural settings because of behaviour, habitat complexity or other confounding factors [23, 24]. It is therefore appealing to measure climatic tolerances from the observed species’ ranges themselves [13, 20, 25].

In particular, to test the hypothesis that wider tolerances increase species’ range size, species’ environmental or habitat niche breadths have generally been measured from the same geographic data as range size was calculated [10, 11]. As predicted, range size consistently increases with species' realized niche breadth [10, 11]. Slayter et al.’s [11] meta-analysis “Niche breadth predicts geographical range size: a general ecological pattern” called for a deeper investigation of the underlying ecological and evolutionary causes. But, in many of the studies compiled by Slayter et al. [11], niche breadth was measured from the same macro-scale data as range size; not independently. While four out of the five studies in Slatyer et al.’s [11] meta-analysis that measured range size and “environmental tolerance breadth” from the same species’ distribution data found the predicted positive relationship, only two out of six studies that used independent data found significant positive relationships (i.e. calcium requirement in Briers [26] and germination temperature in Calosi [21]). The results for “habitat breadth” in Slatyer et al. [11] were similar: 24/26 studies measuring range size and habitat breadth from the same species’ distribution data detected the predicted positive relationship, while only 7/18 studies that used independent data (e.g. microhabitat use, field guide classification, independent census data) did so. It is therefore possible that correlations between climatic niche breadth and range size are inflated when measured from the same distributional data.

Similarly, range-filling, the proportion of a species’ potential range that is occupied, has also been measured directly from the observed range of occupied climates, assuming that species can tolerate the macroclimatic conditions anywhere within this potential range, but not beyond it [27]; but see [24]. Hypotheses have then been proposed regarding the biological factors that could affect the extent of range-filling, e.g. post-glacial migration [27–29], competition (Pearson & Dawson 2003) or colonization ability [30]. Cold- and drought-tolerance have also been measured directly from species’ ranges in order to reach conclusions about potential causal mechanisms explaining variations in range size [17, 31].

Measuring climatic tolerance directly from species geographic ranges to test hypotheses about the biological determinants of species’ ranges rests on the critical assumption that species’ occupied climatic niche are generally closely related to their tolerances [25]. Although physiological climatic limits beyond which any given species cannot survive undoubtedly exist [32], the extent to which they actually impose range boundaries is less clear [33–38]. Most species ranges do not encompass all apparently suitable climates in the surrounding regions [13, 38, 39]. And, terrestrial species’ geographic ranges often fall short of (or extend beyond) the species’ physiological climatic limits [22, 35] such that there are not in climatic equilibrium [40].

If a circumscribed area such as a species’ range is placed randomly on a continent, it will encompass a range of climatic conditions—a realized climatic niche—even if there is no deterministic relationship between that range and the climate on which it was placed. It is already well recognized that the observed niche breadths of widespread species are taken from more locations than those of small-ranged species, which can generate an artefactual positive relationship between range size and niche breadth [41]. Studies have found that the relationship between range size and niche breadth holds even after correcting for this sampling bias [10, 11, 42]. But, what has been underappreciated is that species’ ranges, as well as extant climatic conditions, are highly spatially autocorrelated. Superimposing any spatially autocorrelated surfaces can lead to a correlation between them, even if there is no causal relationship [43–45].

Here, we evaluate the extent to which inter-specific variations in range size would be statistically related to species’ occupied climatic niches (i.e., climatic niche breadth, range filling and niche position) in the absence of any mechanistic connection between these variables. We then ask whether real (i.e. observed) range size–climatic niche relationships are stronger than expected from a randomization of the predictor variable (i.e., climatic niche breadth, range filling or niche position). To do this, we studied the breeding ranges of 3277 non-migratory birds and 1659 mammals occurring in the Americas. Note that, although animals may adjust their behaviour to occupy microclimatic conditions that are very different from the regional average, much of the literature on variation in range size and species’ climatic niche largely rely on coarse-grained environmental and animal distribution data, e.g. [46]. Birds and mammals sometimes show amongst the strongest effect sizes, e.g. Table S3 in [11]. We used macro-scale data on species’ geographic ranges, and we measured (macro)climatic niche characteristics from these. We used a null model that randomizes the spatial variation in climate, while maintaining its spatial autocorrelation [43–45]. This allowed us to examine the extent to which inter-specific variations in range size are related to characteristics of the climatic niche, versus spatial autocorrelation alone. Relationships stronger than the null expectations would be consistent with deterministic effects of climatic tolerances on species ranges.

## Methods

The breeding ranges of 3277 non-migratory birds and 1659 mammals of the Americas were obtained from NatureServe [47, 48] and converted into raster format. We divided the Americas into 4141 equal-area cells of 10,000 km^2^ following Boucher-Lalonde et al. [38], which is roughly the effective resolution of expert-drawn range maps [49]. A cell was considered occupied by a species when its breeding range overlapped the cell at least partly. Mean annual temperature and total precipitation were obtained from the WorldClim database at a 30 arc-seconds resolution [50]. Their means were calculated for each cell.

We measured the properties of species’ occupied temperature and precipitation niche as follows. We measured niche breadth as the range of occupied climate [51–53]. We measured climatic range filling as the realized/potential range size ratio following Svenning & Skov [13] where the potential range is assumed to include all cells with climatic conditions that fall within of conditions that the species’ occupies somewhere within its range. We restricted the potential range to be within the zoogeographical region(s) in which the species occurs (i.e. North American, Mexican, Panamanian, Amazonian, South American and/or Artico-Siberian; Holt et al. 2013), such that climatically suitable areas outside the zoogeographic regions in which the species occurs do not affect the measured potential range (see Fig F in S1 File for results where the potential range is not restricted). We measured niche position as the environmental centroid [54], which is here simply the mean climate of occupied cells. We also calculated the minimum and maximum occupied temperature and precipitation.

We generated null expectations for the empirical relationships between range size and measures of the climatic niche (described below). This was done by randomizing the climatic variables, while maintaining their autocorrelation structure among cells following Chapman [44]. The method is a simple extension of a resampling test, where the climate values are randomly rearranged among cells but where the resampled gradient is constrained to have a similar spatial correlation structure (estimated by empirical variograms) as the real gradients. This procedure removes any causal link between species’ ranges and climate. This could have also been done by placing species’ ranges randomly on the observed climatic gradients in North America[45, 55], but that procedure creates an artificial mid-domain effect [43] and is very sensitive to slight changes in how the null distribution is modelled [56], whereas we found that our conclusions were robust to the details of how the empirical variograms were modelled.

We then assessed how inter-specific variation in range sizes is related to species' climatic niche properties, using general linear models to explore these relationships. We then compared each range size–niche property relationship to the null expectation based on randomized climates (i.e. in which there is no causal link between range size and climatic niches). We also assessed how species’ climatic niche properties (niche breadth, range filling and niche position) covary among species, and we tested whether these correlations were different than those expected from spatial autocorrelation alone. In the literature, niche properties are sometimes correlated, and potential causal mechanisms are then inferred from these correlations [6]. Our aim here was therefore to test whether these correlations are stronger than what we can expect from spatial autocorrelation alone.

To implement Chapman’s [44] procedure to generate randomized gradients in temperature and precipitation, we first quantified spatial autocorrelation in temperature and precipitation in the Americas, at the spatial grain of 10^4^ km^2^. For both temperature and precipitation, we modelled the spatial correlation structure of the data through an empirical variogram, using a powered-exponential function in the 'geoR' package [57] in R [58]. Then, we created a new variable that reproduced this spatial structure using stationary Gaussian Random Fields generated in the 'RandomFields' package [59]. This is a stochastic process by which random numbers are generated and mapped in space. The probability that a randomly generated value is assigned to a particular cell depends on the neighboring values through the variogram model. We then adjusted for the observed mean difference between coastal and inland cells following Chapman [44]. The values for each cell generated by the Gaussian Random Fields where then ranked. In each cell, the rank was replaced with the exact temperature or precipitation that has the same rank order in the real data. This reproduced the general, broad-scale spatial structure of the climatic variables (Fig A in S1 File), but obviously could not account for all its complexity. A detailed description of the algorithm and the R code to generate the simulated null gradients is available in Chapman’s [44] Appendix S3. We created one thousand iterations of these randomized temperature and precipitation surfaces within the Americas to serve as a null model in our analyses. Correlations between the real and randomized variables varied greatly, but they were centered on zero, as were the correlations between the randomized temperature and precipitation variables (Fig M in S1 File).

We then superimposed the observed breeding ranges of North American birds and mammals on each set of randomized climatic variables, and we calculated the niche properties for each species on the randomized climatic surfaces. Since the potential range was restricted to the zoogeographic region(s) in which each species occurs, our null model for the distribution of range filling was calculated by randomizing the climatic variables only within the zoogeographic region(s) in which the species occurs (but see Fig F in S1 File in which we relax this constraint). We then compared the slope and R^2^ of the observed relationships to those obtained from our set of randomizations. Because some randomizations produced climate surfaces that were strongly correlated with observed climate, we repeated all our analyses with a set of 1000 randomizations in which we excluded all randomized climatic surfaces that were correlated with |r| > 0.2, with the real climatic variables. By restricting the randomizations to be uncorrelated with the real variables, we increase our ability to distinguish the real relationships from the null model. These results are presented in Figs I-L in S1 File and were qualitatively similar to those obtained from the full set of randomizations.

To test whether our approach has sufficient power to detect a signal of climate on species’ ranges and whether this is affected by intrinsic (endogenous) spatial autocorrelation (i.e. that arises from population processes such as dispersal), we simulated species’ ranges that are causally related to climate. Specifically, we performed two different simulation models in which we simulated species’ geographic ranges. In these, each species’ range size and climatic niche breadth were constrained by those measured in the real data. Consequently, the simulated niche characteristics were highly correlated to those observed (0.71≤ r ≤ 0.99). In the first simulation model, we probabilistically sampled from all cells that fell within the species’ climatic niche until the species’ range size was reached In this model, any spatial autocorrelation in the range is solely induced from the spatially autocorrelated climatic variables (i.e. exogenous spatial autocorrelation only). In the second simulation model, we additionally modelled strong intrinsic spatial autocorrelation in the species’ range through a simple spreading-dye model. Amongst the cells that fell within the species’ climatic niche, one was randomly seeded, and neighboring cells that fell within the species’ climatic niche were then sequentially selected to be occupied until the species’ range size was attained (if all neighboring cells within the species’ climatic niche were occupied and total range size was not yet attained, a new cell was randomly seeded). For both simulation models, we then compared the range size–climatic niche properties relationships and tested whether they were stronger than those that would have been observed in the 1000 randomizations of the climatic surfaces. This allowed us to test whether, given the variance in the data, a causal relationship between species’ ranges and climate could have been detected in the presence of, versus in the absence of, intrinsic spatial autocorrelation.

## Results

### Correlations among niche properties

Temperature and precipitation niche position are highly positively correlated for mammals (r = 0.68) and for birds (r = 0.64) in the Americas [6], which presumably at least partly reflects the fact that temperature and precipitation in the Americas are highly correlated (r = 0.62). Additionally, temperature and precipitation niche breadth are highly correlated (r = 0.77 for mammals and 0.65 for birds), as well as range filling of the temperature and precipitation niches (r = 0.91) (Table 1). Climatic niche breadth is weakly negatively related to temperature and precipitation niche position (Table 1). The strongest observed correlations were between niche breadth and the minimum temperature or precipitation occupied by the species (Fig N in S1 File). However, all the observed correlations fall either within the range expected from the randomization procedure, or are weaker than expected (Table 1; Figs H-G in S1 File).

**Table 1:** Correlation coefficients from pairwise correlations between the different occupied niche properties for n = 3277 bird and 1659 mammal species within their American breeding ranges. The measured niche properties are the temperature (T) and precipitation (P) breadth, mean position of occupied climates and range filling (i.e., the proportion of cells with suitable climates, in the zoogeographical region(s) in which the species occurs, that fall within the species’ range). The percentile of the simulated coefficients (n = 1000) in which falls the observed coefficient is presented in parentheses. All observed correlations either fell within the range of simulated coefficients or closer to a correlation of zero; none were stronger.

		T breadth	P breadth	T position	P position	T filling	P filling
Birds							
	P breadth	0.65 (24)					
	T position	-0.23 (54)	-0.33 (9)				
	P position	-0.11 (28)	-0.45 (14)	0.64 (96)			
	T filling	0.12 (0)	0.13 (0)	0.15 (82)	0.04 (60)		
	P filling	0.11 (0)	0.03 0)	0.28 (99)	0.12 (79)	0.91 (0)	
	Range size	0.53 (1)	0.40 (0)	0.19 (76)	0.09 (65)	0.66 (34)	0.65 (17)
Mammals							
	P breadth	0.77 (64)					
	T position	-0.27 (43)	-0.19 (16)				
	P position	-0.20 (11)	-0.27 (39)	0.68 (97)			
	T filling	0.22 (0)	0.21 (0)	-0.01 (43)	0.03 (68)		
	P filling	0.17 (0)	0.12 (0)	0.11 (94)	0.14 (97)	0.91 (0)	
	Range size	0.65 (3)	0.59 (1)	0.01 (51)	0.06 (71)	0.61 (93)	0.57 (1)

### Correlations with range size

Since the conclusions were similar for birds and mammals (see S1 File for detailed results), we present the pooled results below. Variations in range sizes are, by definition, entirely explained by the interaction of potential range size (i.e. the spatial extant of climates that are suitable based on occupancy) and range filling. Thus, it is unsurprising that observed range size was positively related to both species’ range filling (r^2^ = 0.41 for temperature and 0.38 for precipitation) and climatic niche breath (r^2^ = 0.34 for temperature and 0.22 for precipitation). Inter-specific variations in range size were only very weakly positively related to niche position (r^2^ = 0.01 for both temperature and precipitation).

**Fig 1:**
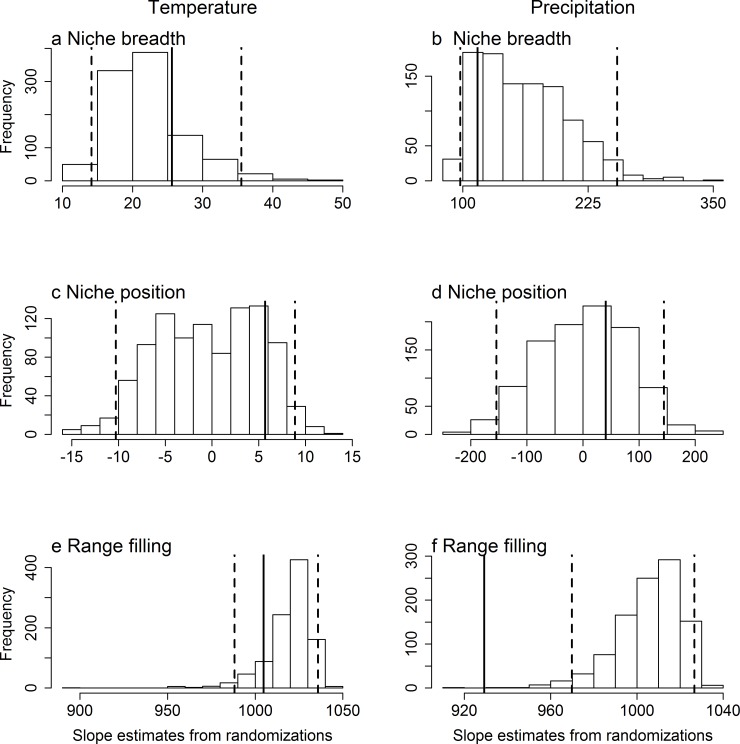
Histograms of expected distribution of slopes between range size and a set of climatic niche characteristics under the null model. The observed slope of the relationship between range size, measured as number of 10^4^-km^2^ cells occupied, and (a, b) niche breadth, (c, d) niche position and (e, f) range filling of the occupied (a, c, e) temperature (°C) and (c, d, f) precipitation (mm) is shown by a vertical solid line. The data are from the American geographic ranges of 3277 non-migratory bird and 1659 mammal species, pooled together. The expected distribution of slopes was generated by randomizing the predictor variable, while maintaining spatial autocorrelation in the climatic variables. The vertical dashed lines represent the 2.5% and 97.5% thresholds of the randomizations.

**Fig 2:**
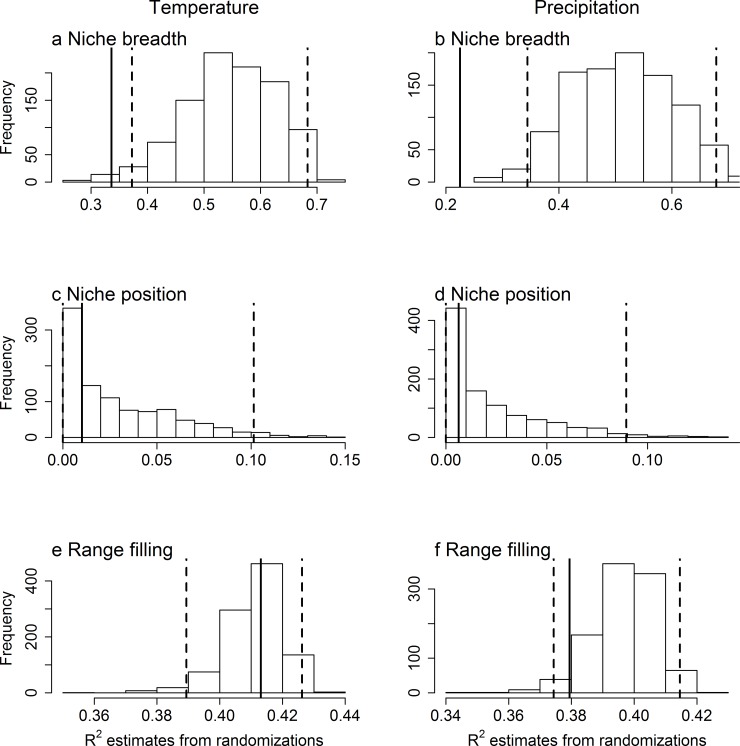
Histograms of expected distribution of coefficients of determination (R^2^) between range size and a set of climatic niche characteristics under the null model. The observed R^2^ of the relationship between range size and (a, b) niche breadth, (c, d) niche position and (e, f) range filling of the occupied (a, c, e) temperature (°C) and (c, d, f) precipitation (mm) is shown by a vertical solid line. The data are from the American geographic range of 3277 non-migratory bird and 1659 mammal species, pooled together. The expected distribution of R^2^ has been generated by randomizing the predictor variable, while maintaining spatial autocorrelation in the climatic variables. The vertical dashed lines represent the 2.5% and 97.5% thresholds of the randomizations.

However, comparing the above results to null expectations, we found that these observed relationships either fell within, or were weaker (closer to zero) than, the relationships expected from a randomization of the predictor variables. Specifically, the observed slopes (Fig 1) and coefficients of determination (Fig 2) were within or below the range expected under a null model in which there is no causal link between range size and the characteristics of the climatic niche, but which conserves the spatial structure in both species’ ranges and climatic conditions. These conclusions were maintained when we analyzed the bird and mammal data separately (Figs B-E in S1 File). When we restricted our set of randomizations to be uncorrelated with the real climatic variables, we also found very similar results (Figs I-L in S1 File). In sum, we cannot reject the null hypothesis that the observed correlation between range size and niche breadth arose from spatial autocorrelation alone.

**Fig 3:**
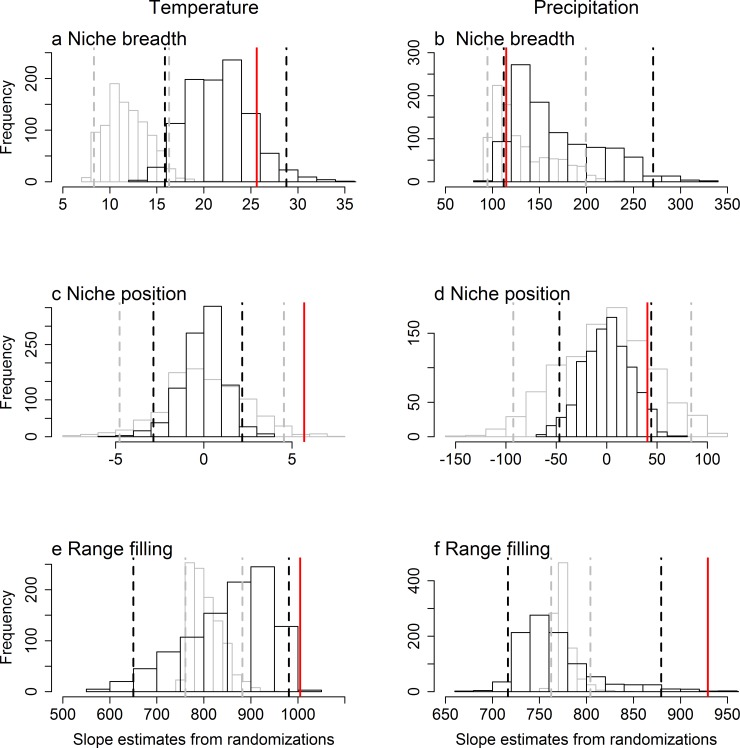
Histograms of expected slopes from power analyses that either incorporate intrinsic spatial autocorrelation (black) or not (gray). Species geographic ranges were simulated to be probabilistically related to temperature and precipitation while maintaining the range size and climatic niche of real species. The observed slope of the relationship between range size, measured in number of cells occupied, and (a, b) niche breadth, (c, d) niche position and (e, f) range filling of the occupied (a, c, e) temperature (°C) and (c, d, f) precipitation (mm) are shown by vertical solid lines. Cells within a species’ climatic niche either had an equal probability of being occupied (i.e., ranges are not necessarily cohesive, shown in gray), or range cohesion, and thereby intrinsic spatial autocorrelation, was modelled through a spreading-dye algorithm (black). For each of the two sets of simulations, we then generated a histogram of the expected distribution of slope values by randomizing the predictor variable, while maintaining its spatial autocorrelation. The vertical dashed lines represent the 2.5% and 97.5% thresholds of the randomizations. This figure is equivalent to Fig 1, but for geographic ranges that have been simulated to be causally linked to macroclimatic variables.

**Fig 4:**
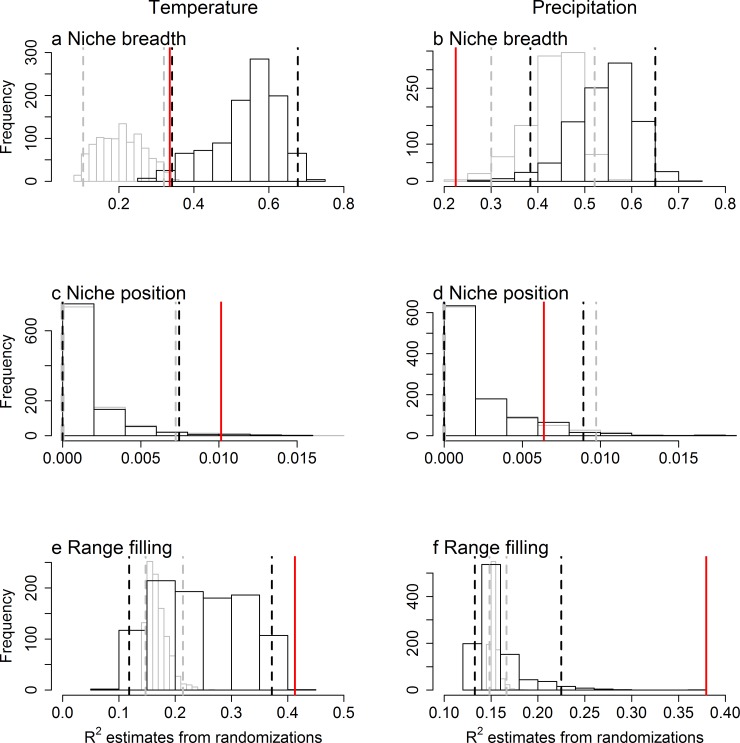
Histograms of expected R^2^ from power analyses that either incorporate intrinsic spatial autocorrelation (black) or not (gray). Species geographic ranges that are probabilistically related to temperature and precipitation and that maintain the range size and climatic niche of real species were simulated. The observed R^2^ of the relationship between range size, measured in number of cells occupied, and (a, b) niche breadth, (c, d) niche position and (e, f) range filling of the occupied (a, c, e) temperature (°C) and (c, d, f) precipitation (mm) are shown by vertical solid lines. Cells within a species’ climatic niche either had an equal probability of being occupied (gray) or intrinsic spatial autocorrelation was modelled through a spreading-dye algorithm (black). For each of the two sets of simulations, we then generated a histogram of the expected distribution of slope values by randomizing the predictor variable, while maintaining its spatial autocorrelation function. The vertical dashed lines represent the 2.5% and 97.5% thresholds of the randomizations. This figure is equivalent to Fig 2, but for geographic ranges that have been simulated to be causally linked to macroclimatic variables.

Our randomization procedures has the statistical power to detect the relationships between range size and climatic niche characteristics as being stronger than expected under our null model only in some cases (Figs 3 and 4). Specifically, when we simulated species’ ranges that are probabilistically determined by climate, while maintaining both range size and the range of climates occupied, the slopes and R^2^ of relationships between range size and temperature niche breadth, temperature niche position as well as temperature and precipitation range filling were higher than those obtained by randomizing these simulated ranges (Figs 3 and 4). However, when we simulated species’ ranges through a spreading-dye model that introduces strong intrinsic spatial autocorrelation, the expected relationships between range size and climatic niche breadth became much stronger. Therefore, the observed slopes and R^2^ of range size as a function of temperature niche breadth becomes undistinguishable from those obtained from the randomization procedure (Figs 3A and 4A). In other words, spatial autocorrelation, and intrinsic spatial autocorrelation in particular, makes causal relationships between range size and species’ climatic niches especially difficult to distinguish from those expected in the absence of causality.

## Discussion

Temperature and precipitation are considered to be among the most important factors determining species’ range boundaries, especially at broad spatial scales [60]. They statistically explain much of the variation in the probability of occupancy of a region by a species [38, 39] as well as inter-specific variations in range size [12, 17]. Consistent with earlier literature, we find that the range size of bird and mammals species in the Americas is positively correlated with their climatic niche breadth, their range filling and their niche position. But, importantly, we found that similarly strong correlations between range size and species’ climatic niche properties (climatic niche breadth, niche position, and range filling) at macro-scales could arise from spatial autocorrelation alone. In the presence of strong spatial autocorrelation, correlations between a species’ range size and the climate niche characteristics that one can extract from this range are expected, regardless of any causal link, therefore providing an inconclusive test of causality.

Of course, species’ ranges could be highly spatially autocorrelated because they are structured by climate in the first place [41, 61]. However, geographic ranges could also be highly spatially autocorrelated because of processes that are independent of climate such as dispersal limitation. Here, we simply argue that the observed correlations between range sizes and niche properties cannot be used to support hypothesized causal links with climatic tolerance. This is because, although the relationships have slopes significantly steeper than zero, they are not stronger than those obtained from a null model that retains spatial autocorrelation. We note that we have only considered a single form of non-independence (spatial autocorrelation), but that other forms likely exacerbate the issue (e.g. phylogenetic relatedness). We argue that, to perform a strong test of the hypothesis that inter-specific differences in climatic niches characteristics cause variations in range size, it is critical that one measures range size and climatic tolerances independently (e.g. in lab measurements). Such tests are less common and the tested correlations have often been weaker or even absent [28, 62–64].

The conflation of environment and geography is a common problem in ecology that canlead to spurious inferences about the importance of deterministic processes in generating spatial patterns in species distributions [25]. It had already been noted that, in the presence of strong spatial autocorrelation, strong relationships between species’ geographic distributions and climate are expected [43–45], making evidence of a strong statistical relationship between the two (e.g., [38, 39]) particularly weak evidence of a causal relationship. Similarly, the conclusion that range size depends on climate largely rests on evidence of a statistical relationship where a species’ range size and climate niche are measured from the same coarse scale data, which also provides particularly weak evidence of causality. When relating range size to potential predictor variables, it is already common practice to account for the fact that wide-ranging species are sampled from more locations than small-ranging species, such that positive relationships are expected in the absence of any causal relationship [41]. We propose that future studies that rely on non-independent data should also explicitly account for spatial autocorrelation in the independent variable (when present).

Our results do raise the possibility that other factors than tolerance of the macro-climate generally explain variations in range size. Slatyer et al. [11] did find that diet breadth positively relate to range size (in addition to tolerance and habitat breadth). It is possible that geographic ranges are determined by a large set of factors. If the importance of these factors varies a lot among species, and through space and time, variations in range size could be at best only weakly predictable by a single predictor. Another (not mutually exclusive) hypothesis is that inter-specific variations in range size are partly driven by the ability of species to maintain high abundances in local communities. Local abundance has a strong negative effect on extinction rates [65] and positive effect on colonization rates of surrounding regions [66] such that range size generally increases with local abundance [67]. But, to rigorously test hypotheses about determinants of range size on equal grounds, and to avoid the risk of inflating the apparent effect of climate and other strongly spatially autocorrelated variables, range size and potential predictor variables need to be measured independently.

We have here shown that the relationship between range size and climate in the range could be explained by the spatial autocorrelation of the climate variables themselves and of the species’ ranges. Spatial autocorrelation greatly inflates the observed relationships between geographic ranges and climate [43, 45]. In sum, while climate obviously excludes many species from occurring in some areas [22], other factors could be more proximately determining species’ ranges, and we would nonetheless observe strong correlations between geographic ranges and climate. Climate could definitely be a major determinant of species’ range and range size, but we caution that some of the correlations commonly presented in the literature do not provide strong evidence of this.

## Supporting Information

S1 File Additional results Figures presenting examples of simulated climatic gradients, results for birds and mammals separately, results where the potential range is unconstrained, randomization distributions for the correlations between niche properties, results where randomizations are constrained to be poorly correlated with real climate and distributions of correlations between real and randomized gradients. (DOCX)
