# Supplementary material for: Spatial Autocorrelation Can Generate Stronger Correlations between Range Size and Climatic Niches Than the Biological Signal — A Demonstration Using Bird and Mammal Range Maps
Source: PLoS One. 2016 Nov 17;11(11):e0166243. doi: 10.1371/journal.pone.0166243 (PMC5113950; doi:10.1371/journal.pone.0166243)
Supplement: S1 File — (DOCX) [file pone.0166243.s001.docx]

Examples of simulated climatic gradients

**Figure A** Observed mean annual temperatures (°C) and total annual precipitations (mm) along with two examples of simulated climatic gradients that randomize observed values while keeping the same spatial autocorrelation structure as in the observed data following Chapman [[44](#_ENREF_44)].

Results from the randomizations of temperature in birds only


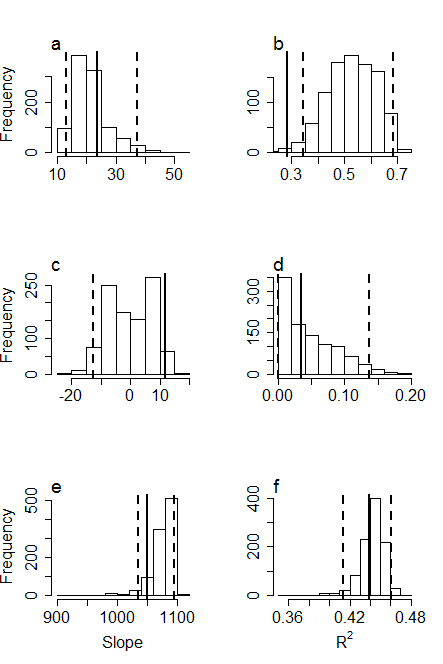


**Figure B** Distribution of (a, c, e) slope and (b, d, f) R^2^ values of bird range size as a function of temperature niche properties obtained from 1000 randomizations of temperatures throughout the Americas (e.g. Figure A). For each randomization, range size was regressed against temperature (a-b) niche breadth, (c-d) niche position and (e-f) range filling. The observed coefficients obtained from 3277 non-migratory bird species are represented by a solid line. Dashed lines represent the threshold of the 5% most extreme values obtained from randomizations.

Results from the randomizations of precipitation in birds only


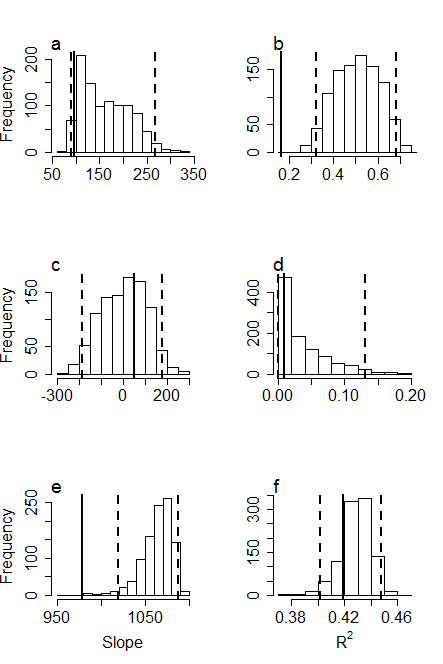


**Figure C** Distribution of (a, c, e) slope and (b, d, f) R^2^ values of bird range size as a function of precipitation niche properties obtained from 1000 randomization of precipitations throughout the Americas (e.g. Figure A). For each randomization, range size was regressed against precipitation (a-b) niche breadth, (c-d) niche position and (e-f) range filling. The observed coefficients obtained from 3277 non-migratory bird species are represented by a solid line. Dashed lines represent the threshold of the 5% most extreme values obtained from randomizations.

Results from the randomizations of temperature in mammals only


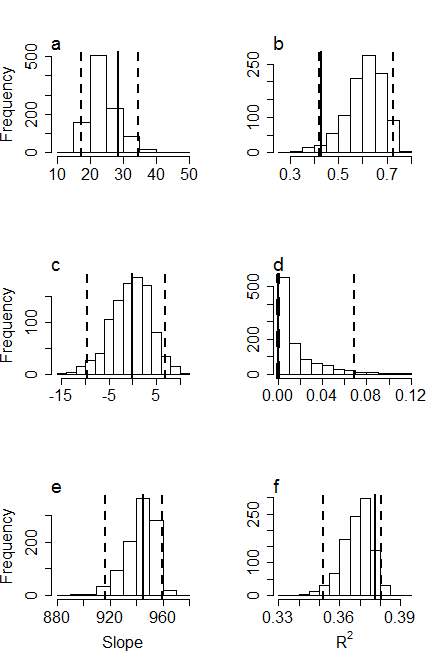


**Figure D** Distribution of (a, c, e) slope and (b, d, f) R^2^ values of mammal range size as a function temperature niche properties obtained from 1000 randomization of temperatures throughout the Americas (e.g. Figure A). For each randomization, range size was regressed against temperature (a-b) niche breadth, (c-d) niche position and (e-f) range filling. The observed coefficients obtained from 1659 non-migratory mammal species are represented by a solid line. Dashed lines represent the threshold of the 5% most extreme values obtained from randomizations.

Results from the randomizations of precipitation in mammals only
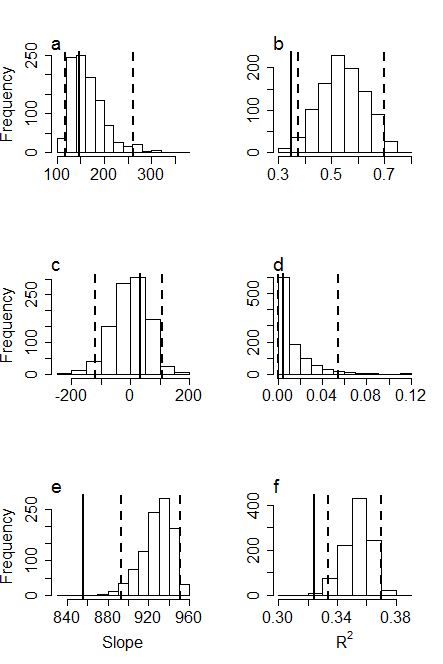


**Figure E** Distribution of (a, c, e) slope and (b, d, f) R^2^ values of mammal range size as a function of precipitation niche properties obtained from 1000 randomization of precipitations throughout the Americas (e.g. Figure A). For each randomization, range size was regressed against precipitation (a-b) niche breadth, (c-d) niche position and (e-f) range filling. The observed coefficients obtained from 1659 mammal species are represented by a solid line. Dashed lines represent the threshold of the 5% most extreme values obtained from randomizations.

All results for the range size–range filling relationship, when the potential range is unconstrained
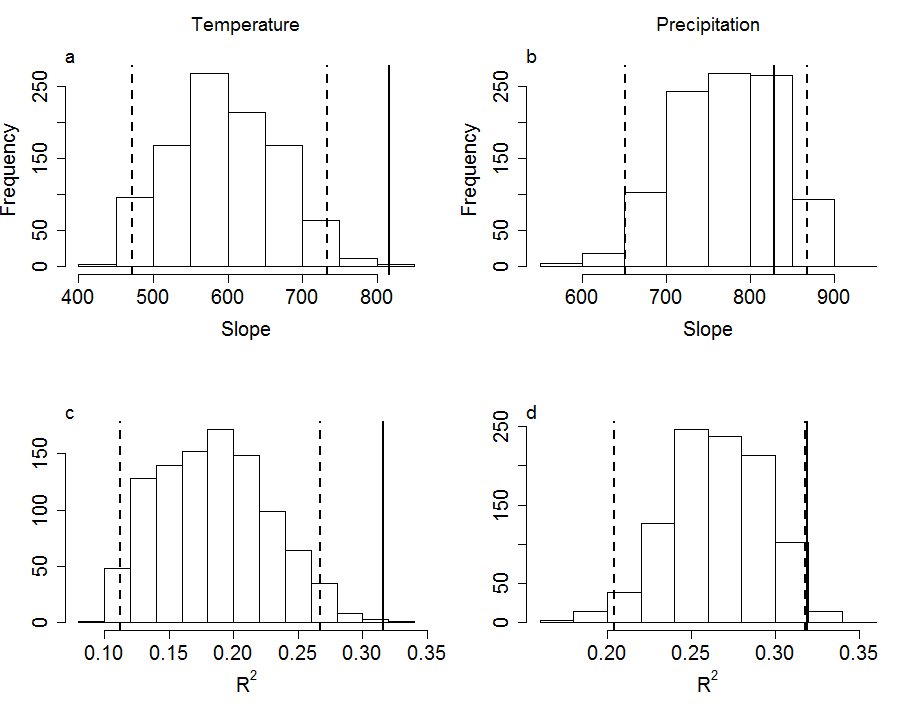
**Figure F** Distribution of (a, b) slope and (c, d) R^2^ values of range size as a function range filling obtained from 1000 randomizations of temperature and precipitation within the Americas. Range filling is the realized/potential range size ratio. Here, the potential range is calculated as all cells within the Americas that fall within the species’ occupied climatic niche. In the main text, the potential range is calculated within the zoogeographic region(s) in which the species occur such that the geographical extent of climatic conditions in distant regions does not affect the potential range (see Figure 1e-f and Figure 2e-f in the main text for comparison). Range filling is calculated for (a, c) temperature and (b, d) precipitation. In each panel, the observed slope obtained from 3277 bird and 1659 mammal species is represented by a solid line. The 5% threshold from the set of randomizations is shown by dashed lines.

Correlations among niche properties (Table 1) compared to expectations from randomizations for birds only


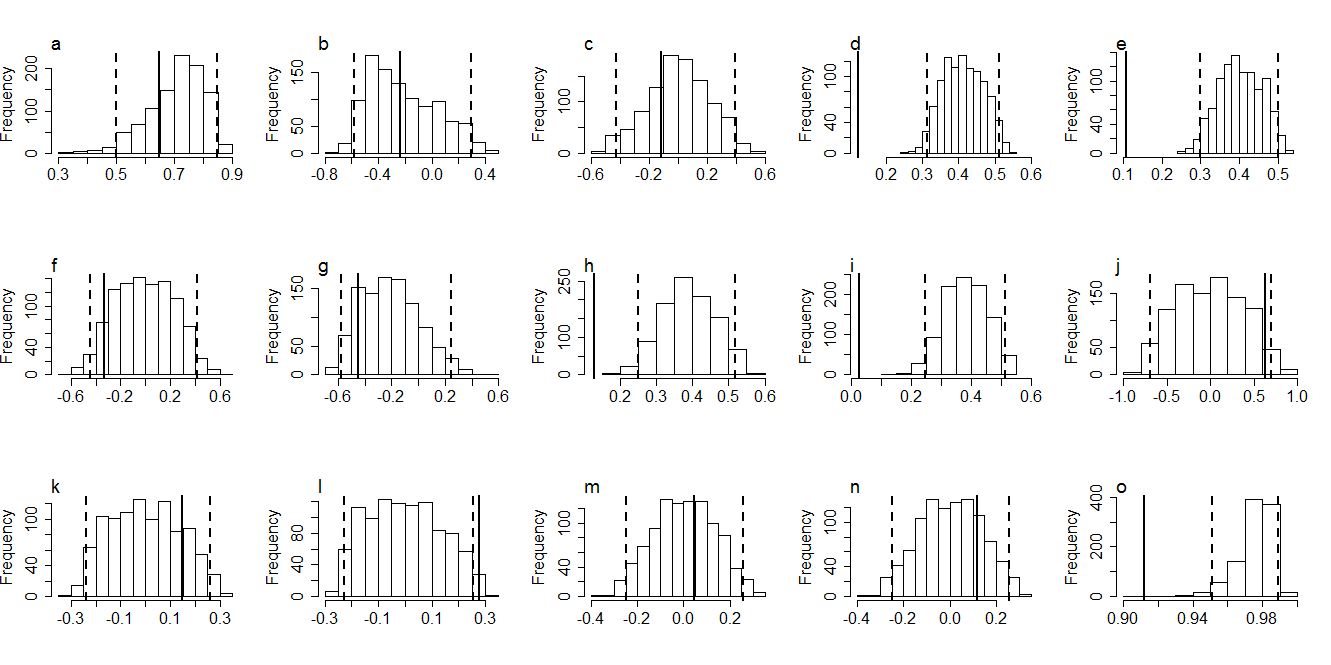


**Figure G** Distribution of correlation coefficients between all pairwise combinations of niche properties of 3277 bird species presented in Table 1 obtained from 1000 randomization of temperature and precipitation within the Americas. The correlations coefficients are presented for (a) temperature and precipitation niche breadth; (b) temperature niche breadth and temperature niche position; (c) temperature niche breadth and precipitation niche position; (d) temperature niche breadth and temperature range filling; (e) temperature niche breadth and precipitation range filling; (f) precipitation niche breadth and temperature niche position; (g) precipitation niche breadth and precipitation niche position; (h) precipitation niche breadth and temperature range filling; (i) precipitation niche breadth and precipitation range filling; (j) temperature and precipitation niche position; (k) temperature niche position and temperature range filling; (l) temperature niche position and precipitation range filling; (m) precipitation niche position and temperature range filling; (n) precipitation niche position and precipitation range filling; and (o) temperature and precipitation range filling. The observed correlation coefficients (presented in Table 1 of the main text) are represented by a vertical solid line. The dashed lines represent the 5% most extreme correlation coefficients obtained from the randomizations (two-tailed).

Correlations among niche properties (Table 1) compared to expectations from randomizations for mammals only


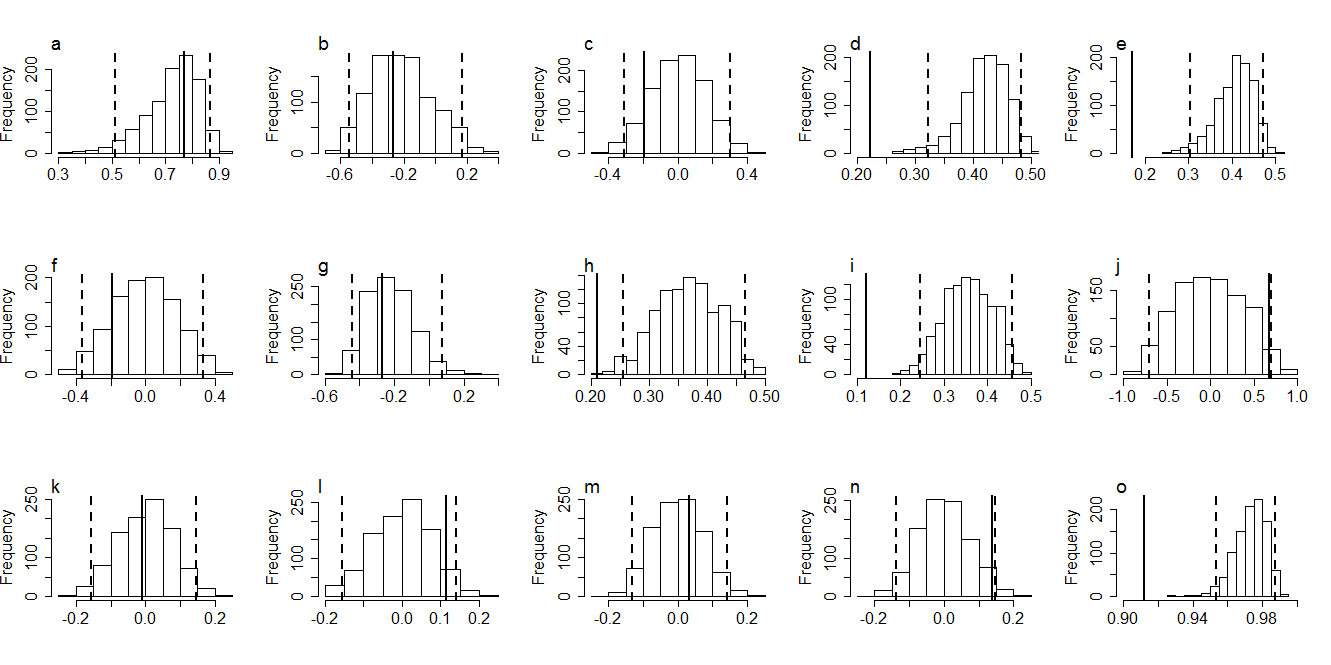


**Figure H** Distribution of correlation coefficients between all pairwise combinations of niche properties of 1659 mammal species presented in Table 1 obtained from 1000 randomization of temperature and precipitation within the Americas. The correlations coefficients are presented for (a) temperature and precipitation niche breadth; (b) temperature niche breadth and temperature niche position; (c) temperature niche breadth and precipitation niche position; (d) temperature niche breadth and temperature range filling; (e) temperature niche breadth and precipitation range filling; (f) precipitation niche breadth and temperature niche position; (g) precipitation niche breadth and precipitation niche position; (h) precipitation niche breadth and temperature range filling; (i) precipitation niche breadth and precipitation range filling; (j) temperature and precipitation niche position; (k) temperature niche position and temperature range filling; (l) temperature niche position and precipitation range filling; (m) precipitation niche position and temperature range filling; (n) precipitation niche position and precipitation range filling; and (o) temperature and precipitation range filling. The observed correlation coefficients (presented in Table 1 of the main text) are represented by a vertical solid line. The dashed lines represent the 5% most extreme correlation coefficients obtained from the randomizations (two-tailed).

Results from a restricted set of randomizations of temperature in birds
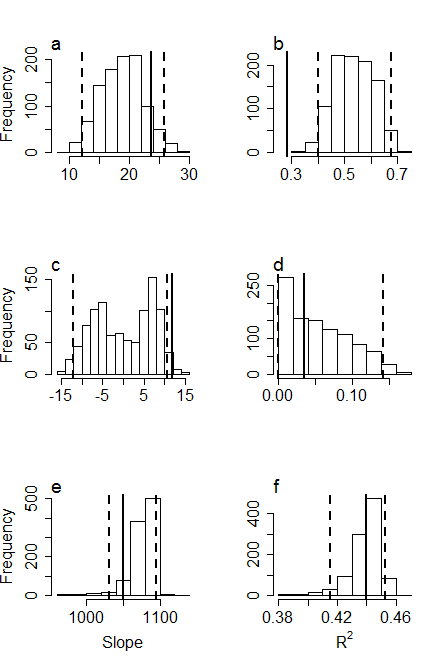


**Figure I** Distribution of (a, c, e) slope and (b, d, f) R^2^ values of bird range size as a function of temperature niche properties obtained from 1000 randomizations of temperatures throughout the Americas. Here, the set of temperature randomizations was constrained to be correlated with the real temperature data with |r| < 0.2. For each randomization, range size was regressed against temperature (a-b) niche breadth, (c-d) niche position and (e-f) range filling. The observed coefficients obtained from 3277 non-migratory bird species are represented by a solid line. Dashed lines represent the threshold of the 5% most extreme values obtained from randomizations (one-tailed for niche breadth and range filling; two-tailed for niche position). This Figure is equivalent to Figure B, but uses a more liberal null model by restricting the set of possible randomizations.

Results from a restricted set of randomizations of precipitation in birds
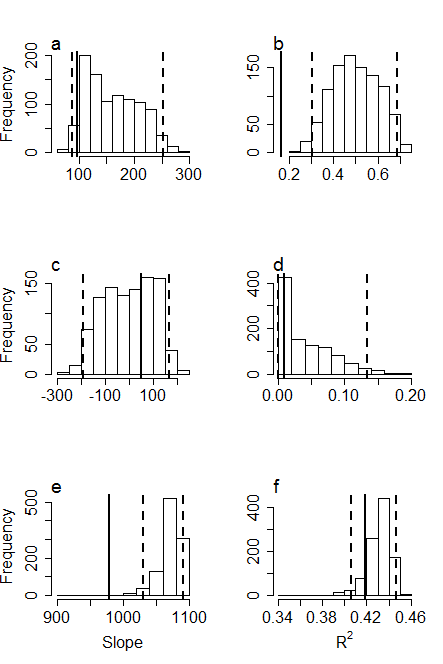

**Figure J** Distribution of (a, c, e) slope and (b, d, f) R^2^ values of bird range size as a function of precipitation niche properties obtained from 1000 randomizations of temperatures throughout the Americas. Here, the set of precipitation randomizations was constrained to be correlated with the real precipitation data with |r| < 0.2. For each randomization, range size was regressed against precipitation (a-b) niche breadth, (c-d) niche position and (e-f) range filling. The observed coefficients obtained from 3277 non-migratory bird species are represented by a solid line. Dashed lines represent the threshold of the 5% most extreme values obtained from randomizations (one-tailed for niche breadth and range filling; two-tailed for niche position). This Figure is equivalent to Figure C, but uses a more liberal null model by restricting the set of possible randomizations.

Results from a restricted set of randomizations of temperature in mammals
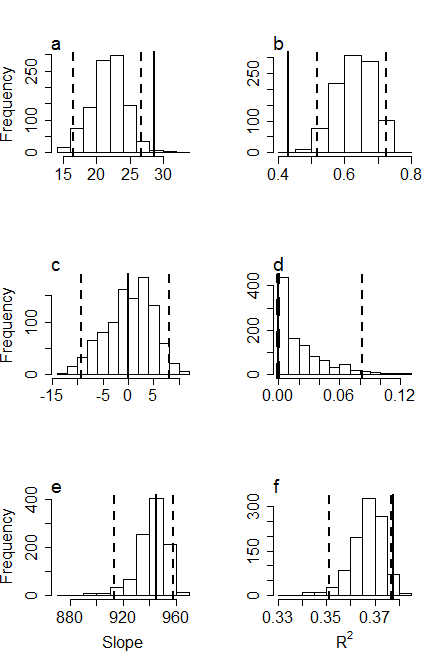


**Figure K** Distribution of (a, c, e) slope and (b, d, f) R^2^ values of mammal range size as a function of temperature niche properties obtained from 1000 randomizations of temperatures throughout the Americas. Here, the set of temperature randomizations was constrained to be correlated with the real temperature data with |r| < 0.2. For each randomization, range size was regressed against temperature (a-b) niche breadth, (c-d) niche position and (e-f) range filling. The observed coefficients obtained from 1659 mammal species are represented by a solid line. Dashed lines represent the threshold of the 5% most extreme values obtained from randomizations (one-tailed for niche breadth and range filling; two-tailed for niche position). This Figure is equivalent to Figure D, but uses a more liberal null model by restricting the set of possible randomizations.

Results from a restricted set of randomizations of precipitation in mammals
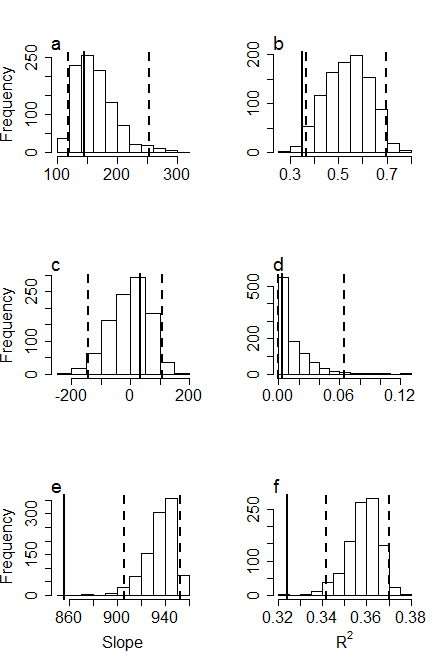


**Figure L** Distribution of (a, c, e) slope and (b, d, f) R^2^ values of mammal range size as a function of precipitation niche properties obtained from 1000 randomizations of precipitation throughout the Americas. Here, the set of precipitation randomizations was constrained to be correlated with the real precipitation data with |r| < 0.2. For each randomization, range size was regressed against precipitation (a-b) niche breadth, (c-d) niche position and (e-f) range filling. The observed coefficients obtained from 1659 mammal species are represented by a solid line. Dashed lines represent the threshold of the 5% most extreme values obtained from randomizations (one-tailed for niche breadth and range filling; two-tailed for niche position). This Figure is equivalent to Figure E, but uses a more liberal null model by restricting the set of possible randomizations.

Correlations between real and randomized gradients


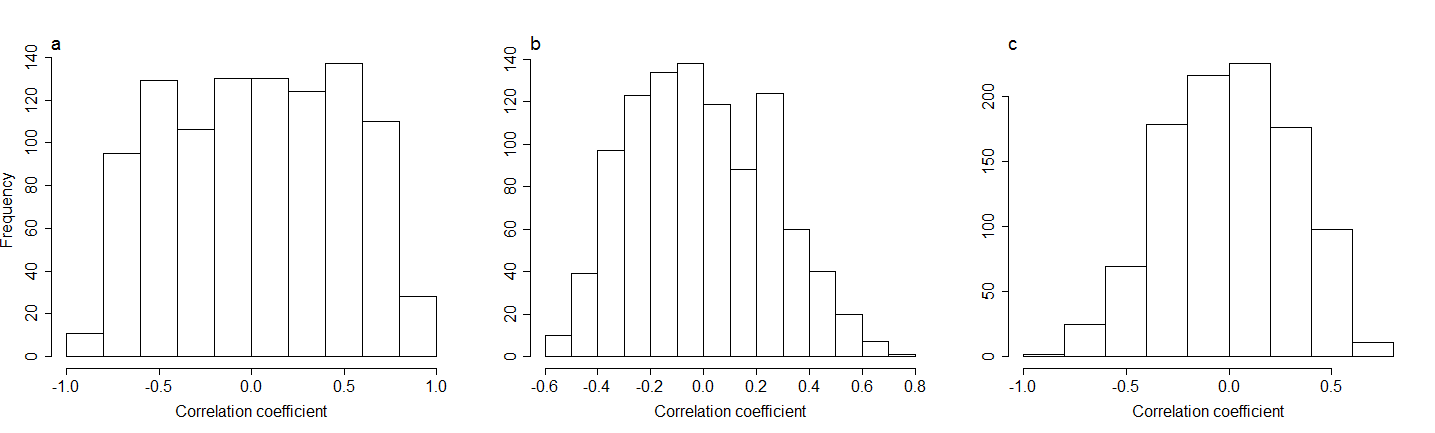


**Figure M** Frequency distribution of the correlation coefficients between (a) real and 1000 iterations of randomized temperature gradients, (b) real and 1000 iterations of randomized precipitation gradients and (c) 1000 iterations of randomized precipitation and temperature gradients.

Niche breadth as a function of the minimum occupied temperature and precipitation

**Figure N** Relationship between the occupied (a) temperature (°C) and (b) precipitation (logarithm of precipitation in mm) breadth and the minimum value occupied by the species. The points represent 3277 non-migratory bird (x) and 1659 mammal (+) species with breeding ranges in the Americas. The fitted regression is represented by the dashed line. These relationships are constrained, since species occupying a low minimum temperature or precipitation can have large niche breadth, whereas species with high minimum temperature or precipitation must have a small niche breadth. The triangular constraints are depicted in light gray. The dark gray lines represent the linear relationships obtained for one thousand randomizations of the independent variable that conserve its spatial structure. The observed correlations (the dashed lines) are stronger than (a) 90.4% and (b) 52.7% of the randomizations.
